# Mobile phone short video use negatively impacts attention functions: an EEG study

**DOI:** 10.3389/fnhum.2024.1383913

**Published:** 2024-06-27

**Authors:** Tingting Yan, Conghui Su, Weichen Xue, Yuzheng Hu, Hui Zhou

**Affiliations:** ^1^Department of Psychology and Behavioral Sciences, Zhejiang University, Hangzhou, China; ^2^Second Affiliated Hospital, School of Medicine, Zhejiang University, Hangzhou, China; ^3^MOE Frontier Science Center for Brain Science and Brain-Machine Integration, Zhejiang University, Hangzhou, China; ^4^Key Laboratory of Novel Targets and Drug Study for Neural Repair of Zhejiang Province, School of Medicine, Hangzhou City University, Hangzhou, China; ^5^The State Key Laboratory of Brain-Machine Intelligence, Zhejiang University, Hangzhou, China

**Keywords:** mobile phone short-form video, attention network, executive control, EEG, addiction tendency

## Abstract

The pervasive nature of short-form video platforms has seamlessly integrated into daily routines, yet it is important to recognize their potential adverse effects on both physical and mental health. Prior research has identified a detrimental impact of excessive short-form video consumption on attentional behavior, but the underlying neural mechanisms remain unexplored. In the current study, we aimed to investigate the effect of short-form video use on attentional functions, measured through the attention network test (ANT). A total of 48 participants, consisting of 35 females and 13 males, with a mean age of 21.8 years, were recruited. The mobile phone short video addiction tendency questionnaire (MPSVATQ) and self-control scale (SCS) were conducted to assess the short video usage behavior and self-control ability. Electroencephalogram (EEG) data were recorded during the completion of the ANT task. The correlation analysis showed a significant negative relationship between MPSVATQ and theta power index reflecting the executive control in the prefrontal region (*r* = −0.395, *p* = 0.007), this result was not observed by using theta power index of the resting-state EEG data. Furthermore, a significant negative correlation was identified between MPSVATQ and SCS outcomes (*r* = −0.320, *p* = 0.026). These results suggest that an increased tendency toward mobile phone short video addiction could negatively impact self-control and diminish executive control within the realm of attentional functions. This study sheds light on the adverse consequences stemming from short video consumption and underscores the importance of developing interventions to mitigate short video addiction.

## 1 Introduction

In today’s rapidly evolving landscape of smartphone technology and widespread mobile internet access, short-form video platforms have become an integral part of people’s daily routines. These platforms, known for their quick and engaging way of sharing information, have amassed a large user base that dedicates significant time to consuming various short video content (Ye et al., 2022). Despite their popularity, there is a noticeable gap in our understanding of the potential addictive tendencies associated with excessive consumption of these mobile short-form videos and their potential impact on cognitive functions. Previous studies on substance abuse have revealed impaired inhibitory control and a pronounced attentional bias toward cues related to the addictive substance (Murphy and Garavan, 2011; Wilcockson and Pothos, 2015; Yang et al., 2019; Bowling et al., 2020; Wilcockson et al., 2021). Investigations into behavioral addictions have also revealed disruptions in the control system that governing attentional processes, and the addicts showed deficits in both attention function and control system (Dong et al., 2017; Wang et al., 2017; Bediou et al., 2018; Kraplin et al., 2020). Similarly, studies in adolescents have shown that excessive use of mobile phones, including short-form video, can lead to social withdrawal and affect normal interpersonal skills (Tateno et al., 2019). Relatedly, excessive use of mobile phone video may have a negative impact on attention and cause certain health risks (Liu et al., 2021; Chen et al., 2022). For example, studies suggest that addicted mobile phone short video users have more attention deficits while watching short-form videos, and have impaired attentional concentration during processing interference (Qi et al., 2022; Zhou et al., 2022). However, the neural mechanism underlying the impact of mobile phone short-form video addiction tendency on the attention is still largely unknown. In fact, investigating how video use impacts neural activity would advance our understanding of how addiction to these stimuli affects attentional processes and ultimately guide more targeted interventions and treatments for individuals struggling with this issue.

Attention plays a pivotal role in cognitive processing with substantial implications for learning and adaptive behavior (Fan et al., 2002; Raz and Buhle, 2006; Petersen and Posner, 2012; Markett et al., 2022). Among many theories on attention, the tripartite-attentional-network theory posited by Petersen and Posner (2012) is well-recognized. The attention network test (ANT) serves as a commonly used paradigm for assessing the individual variability and neural correlates of attentive characteristics based on this tripartite attentional model (Fan et al., 2002, 2005; Neuhaus et al., 2010; Petersen and Posner, 2012; Kustubayeva et al., 2022; Markett et al., 2022). In this model, attention system is divided into three sub-networks, namely, alerting, orienting, and executive control. The alerting network is responsible for sustaining vigilant cognitive states, primarily associated with the left frontoparietal network, including frontal and parietal lobes in conjunction with the thalamus (Posner, 2012). The orienting network is crucial for modulating sensory input and priming actions, prominently engaging the parietal and frontal regions (Proskovec et al., 2018). The executive control network, often referred to as the conflict network, governs self-regulation and conflict resolution arising from cognitive, emotional, and behavioral processes. The prefrontal cortex (PFC), anterior cingulate cortex (ACC), salience network (SN), were assumed playing significant roles in executive control (Fan et al., 2002; Posner, 2012; Bowling et al., 2020; Markett et al., 2022).

In the present study, the ANT task was used to characterize individual’s attentional capability and electroencephalography (EEG) was used to measure corresponding neural activities. In previous studies, theta brainwaves are considered as a key neural oscillation underlying attention function (Hermens et al., 2005; Fan et al., 2007; Xu et al., 2013; Fellrath et al., 2016; Magosso et al., 2021; Asanowicz et al., 2023). Particularly, the association between theta oscillations and cognitive conflict resolution has been a focus of previous research, which suggest an increased frontal theta activity during incongruent trials, compared to congruent ones (Wei and Zhou, 2020). Additionally, studies, including those on meditation, have linked theta and alpha oscillations to internally directed attentional states and positive emotional experiences (Aftanas and Golocheikine, 2001). The primary source of the theta wave activity is suggested to be the prefrontal cortex, including dorsal anterior cingulate cortex, with indications of its potential generation within the upper cortical layers (Botvinick et al., 2004), and the midfrontal cortex’s theta band activities appear to represent a common computational mechanism associated with the need for cognitive control (Cavanagh and Frank, 2014).

Based on previous findings that individuals who are prone to short video addiction show impaired behavioral performance on attention tasks (Chen et al., 2022; Qin, 2022; Ye et al., 2022), we hypothesized that addiction tendencies to short-form videos might have negative impacts on neural activity underlying attentional functioning. Given that previous studies examining the EEG signatures of ANT have highlighted the significance of theta brainwaves for studying attention function (Neuhaus et al., 2010; Fellrath et al., 2016; Magosso et al., 2021; Kustubayeva et al., 2022; Duan et al., 2023), the present study used a time-frequency signal analysis approach to investigate how the neural activity underlying attentional functioning related to addiction tendencies to short-form videos, with a focus on theta band. While addictive tendencies were measured through a short-form video addiction questionnaire (Liu, 2022), participants’ brain activity was recorded using EEG along with the implementation of the ANT (Neuhaus et al., 2010).

## 2 Methodology

### 2.1 Study participants

#### 2.1.1 Recruitment methods

Healthy adult participants were recruited through social media (WeChat) advertisements. Participants who confirmed their use of a mobile phone short-form video app were allowed to sign up for the experiment.

#### 2.1.2 Participant inclusion criteria

Participants needed to meet the following criteria to be eligible for this study:

1. Aged between 18 and 65 years;
2. No diagnosis of neurological disorders or mental illnesses;
3. No substance use (psychotropic drugs or alcohol) in the past month;
4. No severe adverse reactions to stimuli such as flickering lights, auditory stimuli, or electromagnetic radiation;
5. No prior participation in similar EEG studies.

#### 2.1.3 Participant information

A total of 48 healthy participants were recruited for this study, including 13 males and 35 females, with ages ranging from 18 to 33 years (M = 21.80 years, SD = 3.62 years). Participants’ educational backgrounds varied from undergraduate to doctoral degrees.

#### 2.1.4 Ethical approval

This study has obtained approval from the Ethics Committee of Zhejiang University and strictly adheres to ethical principles and regulations for protecting participant privacy. Before the experiment, participants were informed that they would be involved in a study related to short video use, and the general procedure of the experiment was explained to them. Participants were also explicitly informed that they have the right to withdraw immediately from the experiment if they experience any physical or psychological discomfort. All participants provided informed consent prior to participation and received compensation for their time.

### 2.2 Experimental protocol

#### 2.2.1 Overall procedure

**FIGURE 1:**
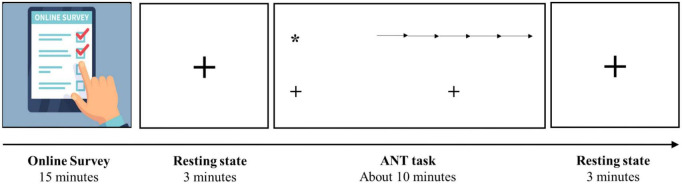
Schematic representation of the experimental protocol.

This research is a part of an umbrella project aiming to address how short-form video watching modulates brain function and structure. Subsequent to the questionnaire completion, two three-minute sessions of eyes-open resting-state EEG recording were obtained before and after the ANT task. Figure 1 schematically represents the experimental protocol, and detailed information about the entire project is available in the supplementary materials (Supplementary Figure 1).

#### 2.2.2 The administration of questionnaires

Participants completed an online questionnaire prior to the EEG experiment. This questionnaire battery encompassed several assessments, shown in the table below (Table 1).

**TABLE 1:** Questionnaires.

No.	Questionnaires
1	Attention Control Scale (ACS, Qin et al., 2022)
2	Barratt Impulsiveness Scale (BIS-II, Patton et al., 1995; Li et al., 2011)
3	Cohen Perceived Stress Scale (CPSS, Yang and Huang, 2003)
4	Depression and Anxiety Brief Symptom Survey (Liu et al., 2013)
5	Five Facet Mindfulness Questionnaire (FFMQ, Baer et al., 2008; Deng et al., 2011)
6	Internet Addiction Test (IAT, Young, 1998)
7	Mind Wandering Questionnaire (MWQ, Mrazek et al., 2013; Ju et al., 2016)
8	Mobile Phone Short-Form Video Addiction Tendency Questionnaire (MPSVATQ, Liu, 2022)
9	Self Control Scale (SCS, Tangney et al., 2004; Tan and Guo, 2008)

To comprehensively delineate addictive behaviors and personality traits, we administered a battery of questionnaires commonly employed in addiction researches. Specifically, the Chinese version of the mobile phone short-form video addiction tendency questionnaire (MPSVATQ) was utilized to assess the tendency toward mobile phone short-form video addiction. This unidimensional instrument, derived from the Internet Addiction Test (IAT), yields higher scores indicative of greater addiction tendency and lower scores indicative of less tendency toward addiction. Its reliability and validity have been rigorously evaluated, yielding satisfactory outcomes (Liu, 2022).

Additionally, the Attention Control Scale (ACS) was employed to appraise attentional capacities, while the Barratt Impulsiveness Scale (BIS-II) was utilized to assess impulsivity. The Cohen Perceived Stress Scale (CPSS) served to gauge subjective stress perceptions, and the Depression and Anxiety Brief Symptom Survey was utilized to evaluate symptoms of depression and anxiety. Furthermore, the Five Facet Mindfulness Questionnaire (FFMQ) was administered to assess mindfulness levels, and the Mind Wandering Questionnaire (MWQ) was used to evaluate levels of mind wandering during daily activities. Lastly, the self-control scale (SCS) was employed to assess individuals’ capacity for behavioral regulation. These measures were undertaken to explore potential associations with short-form video addiction.

#### 2.2.3 The administration of ANT task

**FIGURE 2:**
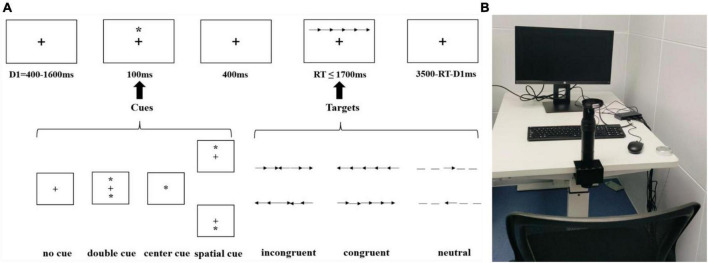
**(A)** the ANT schematic diagram, and **(B)** the experimental setting.

When performing the ANT task (Fan et al., 2002; Figure 2A), participants were seated with their chin secured using a chinrest apparatus (Figure 2B).

Stimuli were presented using a custom program developed with Psychtoolbox^1^ and participants’ behavioral responses were recorded via a standard keyboard placed on a computer desk. A total of 192 trials were administered, with 48 trials for each of the four cue types: no cue, center cue, spatial cue, and double cue. Under each cue condition there was 16 trials of neutral, congruent, and incongruent target stimuli, respectively. Throughout the experimental session, a fixation was consistently displayed at the center of the screen. During each trial, a fixation was first displayed with a duration randomly selected from 400 to 1,600 ms. And then, a cue stimulus with a duration of 100 milliseconds was displayed. This cue appeared either above or below the central crosshair (spatial cue), above and below the screen center (double cue), at the central position (central cue), or was absent (no cue). The central cue and double cue provide information about the timing of the impending target presentation (alerting effect), while the spatial cue also conveyed information about the location of the forthcoming target (orienting effect). The target stimulus consisted of five arrows or lines arranged horizontally, presented either above or below the central crosshair. It would disappear once the participants made a judgment, with a maximum presentation duration of 1,700 milliseconds. Participants were instructed to identify the direction of the central arrow when the target stimulus displayed, irrespective of whether the surrounding flanking arrows were straight (neutral), in a consistent orientation, or in an inconsistent orientation (response suppression). Specifically, participants were required to press the “F” key if the middle arrow in the target stimulus pointed left and the “J” key if it pointed right. A post-response interval with variable duration was implemented to ensure a consistent total trial duration of 4,000 milliseconds for each trial. Participants were first required to complete a series of practice trials of the ANT task.

Only individuals who attained an accuracy rate of 85% or higher in the practice trials were deemed eligible to proceed to the formal ANT task. Each practice session contains 12 trials. It should be noted that no participant failed. There are 3 participants could practice up to three times, others needed only one session to achieve an accuracy rate exceeding 85%. Participants were instructed to respond with both speed and accuracy, and no feedback was provided. The ANT experiment lasted approximately 10 min.

### 2.3 EEG signal acquisition and preprocessing

#### 2.3.1 Acquisition of EEG signal

The entire experimental procedure was conducted at a shielded EEG Laboratory room. A 64 channel electrode caps (Neuroscan Quick-cap, Neuroscan Inc., Sterling, VA, USA) was used and the international 10–20 system for electrode placement was employed. The EEG signal was sampled using a Neuroscan Synamps2 amplifier at 1,000 Hz. Concurrently, vertical and horizontal ophthalmic signals were collected. Generally, these signals are collected to potentially integrate them into subsequent offline EEG analysis. Although these signals can be omitted during the data processing phase if deemed unnecessary, we collect them to ensure the comprehensiveness of our signal acquisition. Impedances were ensured to remain below 10 kΩ before recording.

#### 2.3.2 Preprocessing of EEG data

Offline preprocessing was performed on the EEG data obtained during the ANT task and the resting-state, utilizing EEGlab^2^. For the EEG data of the ANT task, preprocessing steps involved: (1) elimination of redundant electrodes (CB1, CB2) and the vertical and horizontal electrooculograms (VEO and HEO); (2) down-sampling the signal to 250 Hz; (3) selection of bilateral mastoid electrodes (M1, M2) as references; (4) bandpass filtering with the band range of 0.1–35 Hz; (5) notch filtering at 49–51 Hz to remove powerline interference; (6) segments of signal were acquired by cutting continuous signal from −200 to 1,300 ms with respect to cue onsets; (7) employing Independent Component Analysis (ICA) to mitigate ocular and cardiac artifacts. For resting-state EEG data, preprocessing didn’t encompass signal segmentation, other preprocessing steps were the same.

#### 2.3.3 Time-frequency transformation and the spectral analysis of EEG data

For the EEG data of ANT task, the preprocessed data were subjected to Short-Time Fourier Transform (STFT) analysis to delineate their temporal and spectral characteristics (using the EEGLAB toolbox). That is, the EEG signals related to three sub-networks of the attention network: alerting, orienting, and executive control, are transferred to the time-frequency domain, which facilitate us to analyze the time-frequency characteristics of the neural activity. Specifically, a window size of 0.5 s was chosen to capture salient temporal features. In STFT processing, the choice of time window duration impacts the preservation of frequency content within the signal. Opting for a longer time window facilitates the retention of low-frequency components, whereas selecting a narrower time window enhances the preservation of high-frequency components. Given the focus of this study on the theta (4–8 Hz) frequency band, the selection of a 0.5-s time window is deemed advantageous for preserving the characteristic features of signals within this frequency range. Regarding the time window of interest (TWI), it was determined based on ANT task design and previous findings (Neuhaus et al., 2010; Chang et al., 2015; Kustubayeva et al., 2022). The selection of regions of interest (ROIs) is based on brain regions commonly examined in studies using ANT tasks (Fan et al., 2002; Posner, 2012; Proskovec et al., 2018; Bowling et al., 2020; Markett et al., 2022).

For the alerting function, the TWI encompassed 650–800 ms following the onset of cue stimuli. In the spectral domain, the theta frequency range (4–8 Hz) was designated as the frequency window of interest (FWI). Mean amplitude calculations were performed on electrodes located at the parietal region (P5, P3, P1, Pz, P2, P4, P6), the parieto-occipital region (PO5, PO3, POz, PO4, PO6), and the occipital region (O1, Oz, O2). Specifically, the mean amplitude was calculated by adding the mean amplitudes from selected electrode points of interest, after obtaining the mean amplitudes from all trials. The log mode is used to perform the baseline correction. Analogous methodologies were applied to the orienting function, with the TWI set at 600–800 ms following the cue stimulus onset, and the same theta frequency range (4–8 Hz) was selected. Electrode clusters comprising the parietal, parieto-occipital, and occipital regions were analyzed accordingly. For the executive control function, the TWI extended from 600 to 900 ms following the appearance of cue stimuli, and the same theta frequency range was selected. The ROIs for amplitude analysis encompassed frontal electrodes (F5, F3, F1, Fz, F2, F4, F6), central frontal electrodes (FC3, FC1, FCz, FC2, FC4), central electrodes (C5, C3, Cz, C2, C4), parietal electrodes (P5, P3, P1, Pz, P2, P4, P6), and parieto-occipital electrodes (PO5, PO3, POz, PO4, PO6). Regarding the analysis of the resting state EEG data, we utilized the Fast Fourier Transform (FFT) analysis. The absolute power values in decibels (dB) obtained through FFT were used as the index of neural oscillation. Specifically, we focused on the theta band (4–8 Hz), and the chosen brain region was same as that for the task data analysis.

### 2.4 Statistical analysis

#### 2.4.1 Behavioral data analysis

First, we analyzed the pairwise correlation between all the measured questionnaires. Next, for the analysis of behavioral data, a 4 × 3 repeated-measures analysis of variance (RMANOVA) was conducted separately for mean reaction time (RT) and accuracy (ACC), with cue conditions (no cue, center cue, double cue, or spatial cue) and target conditions (congruent, neutral, or incongruent) treated as within-subject factors.

To investigate the association between the efficiency of the three attention sub-networks and the propensity for short video addiction, correlation analyses were performed at both the behavioral and neurophysiological levels. Specifically, regarding the behavioral data analysis, the scores of 3 sub-networks of ANT were correlated with MPSVATQ, respectively. In the ANT task, the scores of three sub-networks of attention were quantized as below:

Alerting Effect = RT of “no cue” condition − RT of “double cue” condition;
Orienting Effect = RT of “center cue” condition − RT of “spatial cue” condition;
Inhibitory Effect = RT of “incongruent” condition − RT of “congruent” condition.

#### 2.4.2 The relationships between short-video addiction tendency and EEG metrics during the ANT task

Regarding the neuroelectric data analysis, we conducted a thorough examination of the correlation between the neural oscillation index reflecting the three sub-networks and the MPSVATQ cross diverse brain regions. The calculation of neural oscillation indexes reflecting the three sub-networks were similar to that in the behavior analysis. Specifically, the indexes of neural oscillation were calculated by replacing RT with mean theta power in the formula above. In addition, gender, age, anxiety and depression scores were used as covariates. Since pervious research revealed that the persons with Facebook addiction have weaker self-control (Blachnio and Przepiorka, 2016), and a significant negative correlation between MPSVATQ and SCS (see below) was also found in the current study, we further conducted correlation analyses to study the relationship between SCS and the neural oscillation indexes reflecting three attentional sub-networks across different brain regions. Finally, we meticulously analyzed the resting-state EEG data to demonstrate whether the correlation between MPSVATQ and neural oscillation index is task-dependent.

In all analytical procedures, a *p*-value smaller than 0.05 was considered as significant. To address the issue of Type I errors arising from multiple comparisons, Bonferroni correction at appropriate junctures was applied. The statistical analysis was executed using SPSS 22.0 and MATLAB 2013b.

## 3 Results

### 3.1 Demographic information and questionnaire results

Demographic information was displayed in Table 2. A significant positive correlation was found between MPSVATQ and IAT scores, and there was a significant negative correlation between the scores of MPSVATQ and both ACS and SCS scores (Table 3). While we administered additional questionnaires for measurement purposes, we solely present herein the results that demonstrate significant associations with MPSVATQ, detailed information is in the supplementary materials (Supplementary Table 1).

**TABLE 2:** Mean and standard deviation of age and scores of each questionnaire scale (*N* = 48).

	Age	MPSVATQ	IAT	Anxiety	Depression	SCS	ACS	BIS-II	MWQ	CPSS	FFMQ
M	21.48	50.77	54.83	12.35	10.71	14.88	3.10	36.53	18.56	40.67	122.71
SD	3.16	11.87	11.17	3.06	3.40	2.19	0.60	7.00	4.11	6.14	9.32

MPSVATQ, mobile phone short video addiction tendency questionnaire; IAT, Internet Addiction Test; SCS, Self Control Scale; ACS, Attention Control Scale; BIS-II, Barratt Impulsiveness Scale; MWQ, Mind Wandering Questionnaire; CPPS, Cohen Perceived Stress Scale; FFMQ, Five Facet Mindfulness Questionnaire.

**TABLE 3:** Pairwise correlation results between the four questionnaires (*N* = 48).

	MPSVATQ	IAT	SCS	ACS
MPSVATQ	–			
IAT	0.390**	–		
SCS	−0.320*	−0.726**	–	
ACS	−0.310*	−0.443**	0.268	–

(*p < 0.05, **p < 0.01).

### 3.2 Behavioral results

#### 3.2.1 Efficiency of the three attentional networks

**FIGURE 3:**
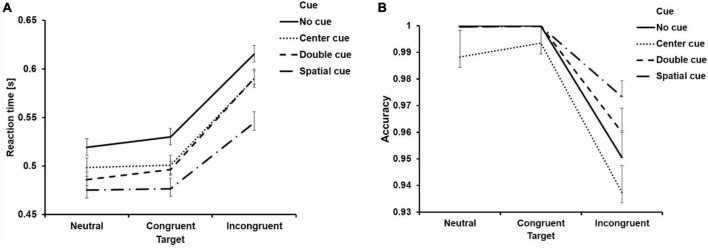
Behavioral results. Mean reaction time from correct trials **(A)** and accuracy **(B)** as a function of cue and target condition. Error bar represents the standard error of mean.

First of all, the extreme values exceeding 3 standard deviations from the mean were replaced by the corresponding average value (Fan et al., 2002; Neuhaus et al., 2010), and the descriptive statistics were displayed in Table 4 and Figure 3.

**TABLE 4:** Descriptive statistical results of reaction time (RT) and accuracy (ACC).

	Target					
	Neutral target		Congruent target		Incongruent target	
Cue	RT (M ± SD) ms	ACC (M ± SD) %	RT (M ± SD) ms	ACC (M ± SD) %	RT (M ± SD) ms	ACC (M ± SD) %
No cue	519.89 ± 5.60	99.997 ± 0.02	529.87 ± 6.57	99.989 ± 0.053	615.24 ± 8.47	95.052 ± 6.69
Center cue	498.31 ± 6.06	98.828 ± 2.47	501.07 ± 6.73	99.349 ± 1.93	589.99 ± 7.02	93.750 ± 6.70
Double cue	485.88 ± 4.45	99.957 ±	496.55 ± 6.65	99.995 ± 0.03	590.25 ± 8.50	96.002 ± 5.97
Spatial cue	475.24 ± 5.58	99.957 ± 0.15	476.73 ± 6.48	99.976 ± 0.10	544.68 ± 7.50	97.331 ± 4.42

Then, we carried out a 4 (cue condition: no cue, center cue, double cue, spatial cue) × 3 (target type: neutral, congruent, incongruent) ANOVA on the RT and ACC. Regarding RT, both main effect of cue type and target type were significant (Table 5). Additionally, the interaction effect between cue and target type was also found to be significant (Table 5). Follow-up analysis indicated that the RT for the “no cue” condition exhibited the longest duration, which was longer than RTs for the “center cue” (adjusted *p*_(Bonferroni correction)_ < 0.001), “double cue” (adjusted *p* < 0.001), and “spatial cue” (adjusted *p* < 0.001) conditions, with the “spatial cue” condition displaying the shortest RT (Table 4). RT for the incongruent condition was significantly longer than that for the congruent condition (adjusted *p* < 0.001).

**TABLE 5:** ANOVA results of ANT network for reaction time (RT) and accuracy (ACC).

Measure	Factor	*F*-value	*p*-value	Partial η^2^
RT	Cue	*F*(3, 141) = 70.20	<0.001	0.60
	Target	*F*(2, 94) = 134.40	<0.001	0.74
	Cue × target	*F*(6, 282) = 4.59	<0.001	0.09
ACC	Cue	*F*(3, 141) = 10.27	<0.001	0.18
	Target	*F*(2, 94) = 48.47	<0.001	0.51
	Cue × target	*F*(6, 282) = 2.60	= 0.018	0.05

The ANOVA on ACC displayed similar results, with significant main effect of cue type and target type (Table 5). The interaction effect between cue and target type was also found to be significant (Table 5). Follow-up analysis indicated that the ACC for the “center cue” condition was lower than ACC for the “no cue” (adjusted *p* = 0.046), “double cue” (adjusted *p* = 0.001), and “spatial cue” (adjusted *p* < 0.001) conditions, with the “spatial cue” condition displaying the highest accuracy (Table 4). ACC for the incongruent condition was significantly lower than that for the congruent condition (adjusted *p* < 0.001).

#### 3.2.2 Correlation analyses: MPSVTQ and RT of ANT

As for the relationship between the efficiency of three attention sub-networks and short-form video addiction tendency, no statistically significant correlation was observed (all *p* > 0.05). Detailed results can be found in the supplementary materials (see Supplementary Table 2).

### 3.3 EEG results

The EEG data of the ANT task of 3 participants were excluded because the participants did not strictly comply with the instructions. Specifically, during the EEG experiment, these participants did not maintain stable head positions, resulting in excessive head movement artifacts in the EEG signals. Therefore, 45 participants were included in the analysis.

#### 3.3.1 Non-significant correlation between addiction tendency and EEG metrics associated with ANT subcomponents

We found no significant correlation between MPSVATQ scores and neural oscillation indexes reflecting the alerting network, orienting network, or executive control network (all *p* > 0.05). The indexes of neural oscillation were calculated by replacing RT with mean theta power in the formula as follows: Alerting Effect = power of the “no cue” condition—power of the “double cue” condition; Orienting Effect = power of the “center cue” condition—power of the “spatial cue” condition; Inhibitory Effect = power of the “incongruent” condition—power of the “congruent” condition. Specific details of these results can be found in the supplementary materials, Supplementary Table 3.

#### 3.3.2 Negative correlation between addiction tendency and the theta power when cognitive control is demanded

**FIGURE 4:**
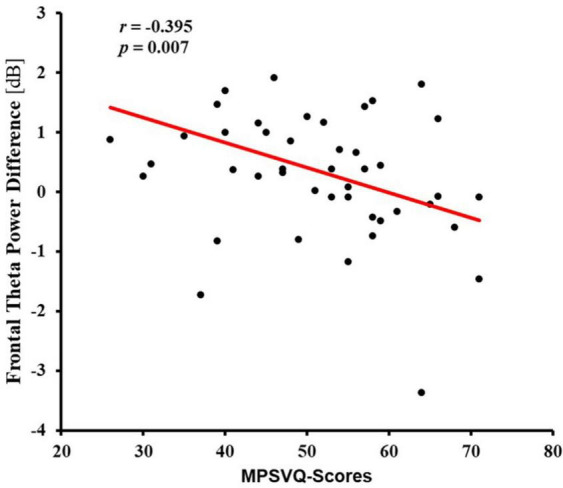
Scatter plot for correlation between addiction tendency (MPSVATQ score) and frontal theta power difference between incongruent target and neutral target of the ANT task.

**FIGURE 5:**
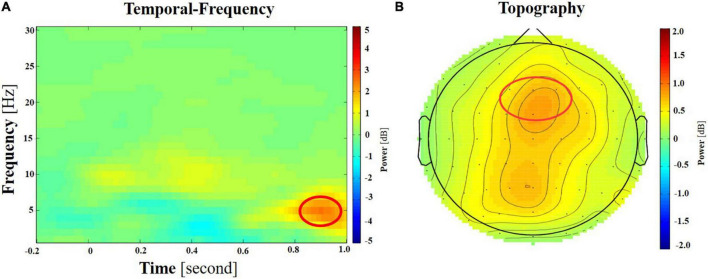
**(A)** The spectrogram of the difference between incongruent target and neutral target at frontal region (illustrated by electrode Fz here). The bands of interest, theta (4–8 Hz), are circled in red. **(B)** Topography of theta power difference between incongruent and neutral targets of the ANT task, during a time window of 600–900 ms post the cue onset.

A noteworthy negative correlation was only found between the activity of the frontal region and MPSVATQ scores when assessing the theta power under incongruent target condition, with neutral target conditions serving as a control baseline condition (*r* = −0.395, *p* = 0.007, Table 6 and Figure 4). These EEG findings were further elaborated in Figure 5. Despite controlling for gender, age, anxiety, and depression, this negative associations remained significant (for more details please refer to the supplementary materials, Supplementary Table 4). Although no significant negative correlation was found between the activity of the central frontal region, central region, parietal region, and parietal occipital region with MPSVATQ scores when assessing theta power under the incongruent target condition, with neutral target conditions serving as a control baseline, the correlation trend is negative.

**TABLE 6:** Correlation coefficients between MPSVATQ and theta power difference between incongruent target and neutral target (*N* = 45).

Brain regions	*R*	*p*
Frontal region (F5, F3, F1, Fz, F2, F4, F6)	−0.395	0.007
Middle frontal region (FC3, FC1, FCz, FC2, FC4)	−0.280	0.063
Central region (C5, C3, Cz, C2, C4)	−0.229	0.130
Parietal region (P5, P3, P1, Pz, P2, P4, P6)	−0.014	0.926
Parietal occipital region (PO5, PO3, POz, PO4, PO6)	−0.118	0.439

The time window for theta power difference is defined as 600–900 ms following cue presentation.

The correlation analysis aimed to examine the relationship between SCS and prefrontal region activity revealed no significant results between SCS scores and the theta power under incongruent target condition, with neutral target conditions serving as a control baseline condition (*p* > 0.05, please refer to supplementary materials, Supplementary Table 5).

To demonstrate whether the correlation between MPSVATQ and neural oscillation is task-dependent, we conducted an analysis of resting state EEG data. The results showed that no significant correlation was found between theta power of two resting-state EEG sessions and MPSVATQ within the prefrontal region (*p* > 0.05, please refer to supplementary materials, Supplementary Table 6). The theta power within the prefrontal region between the two resting-state EEG sessions was not significantly different [*t* (43) = 1.42, *p* = 0.888].

## 4 Discussion

The principal aim of this study was to explore the complex relationship between mobile phone short video addiction tendency and attention, with focus on alerting, orienting, executive control sub-networks assessed by the attention network test (ANT). Correlation analyses revealed a significant negative relationship between MPSVATQ and self-control, in addition, a significant negative correlation was also found between MPSVATQ and the theta power difference under incongruent minus neutral target condition of the ANT, suggesting that individuals with a proclivity for mobile phone short video addiction may encounter challenges related to executive control and self-control abilities.

In this study, the ANT task evaluates three sub-networks: alerting, orienting, and executive control. Executive control assessed by ANT is quantified by the Inhibitory Effect, which was calculated as the difference in response times (RT) between “incongruent” and “congruent” conditions. This method aligns with measurements used in established executive control tasks like the Stroop, Flanker, Go-Nogo, and Stop-signal tasks. Importantly, while ANT parallels the Flanker task in measuring executive control, it also assesses alerting and orienting functions, providing a comprehensive assessment of attentional networks. Contrary to expectations, we did not find a significant correlation between MPSVATQ and ANT’s behavioral performance. It is possible that the simplicity of the task may result in insufficient variation in performance, thereby obscuring the detection of any potential correlations.

However, a significant correlation was observed between MPSVATQ and theta power difference under incongruent minus neutral target condition in the frontal area. This finding aligns with previous studies showing significant differences between addicts and non-addicts in EEG measures but no significant difference in behavioral performance (Zhou et al., 2022). Importantly, after controlling for variables such as anxiety, depression, age, and gender, the association between MPSVATQ and theta power in the frontal area was still significant. These findings highlight the importance of the prefrontal area in executive control, which is consistent with previous studies on addiction. For example, a recent research revealed lower perceptual sensitivity and hypoactivations during inhibitory control in cognitive control regions (e.g., anterior prefrontal cortex, dorsolateral prefrontal cortex, supplementary motor area), which was associated with task performance and heroin use severity measures in individuals with heroin addiction (Ceceli et al., 2023). Previous investigations into behavioral addiction also highlighted the significant involvement of the prefrontal lobe in executive control. Specifically, research on Internet addiction shows that the slow-wave activities in the frontal areas were correlated with the commission error rate in the Go/NoGo task in the internet disorder group (Qi et al., 2022). An earlier study has shown prefrontal cortex may be involved in the circuit modulating impulsivity, and its impaired function may relate to high impulsivity in adolescents with Internet Game Addiction (IGA), which may contribute directly to the Internet addiction process (Ding et al., 2014). Studies have concluded that compared to healthy controls, gaming addicts have poorer response-inhibition and emotion regulation, impaired prefrontal cortex functioning and cognitive control (Kuss et al., 2018). The significant negative correlation between MPSVATQ and theta power difference under incongruent minus neutral target condition in the frontal area suggests the use of short video has negative impact on the neural processing underling executive control, even in the absence of observable differences in specific behavioral tasks. Moreover, the theta power of resting state EEG data, measured before and after the ANT task, did not show significant correlation with MPSVTQ, indicating the independence of the task-related findings in this study. Despite the lack of direct associations in certain behavioral manifestations, the identification of relationship between MPSVTQ and theta power reflecting the executive control network in the frontal area highlights the essential role of frontal brain regions in short-form video addiction, providing valuable insights for prospective intervention and prevention strategies. Prior research has prominently underscored the relevance of theta oscillations to cognitive conflict resolution, particularly evident in augmented frontal theta activity during incongruent trials relative to congruent trials (Wei and Zhou, 2020). This observation implies a pivotal role of theta band oscillations in processes associated with conflict detection, monitoring, and resolution, where heightened theta event-related synchronization (ERS) magnitude correlates with escalated engagement of cognitive control (Nigbur et al., 2011; Cavanagh and Frank, 2014). The findings of this study revealed a notable negative correlation between MPSVATQ and theta power difference under incongruent minus neutral target condition. Consequently, it is postulated that heightened propensity toward mobile phone short-form video addiction may correspond to diminished executive control capacities. Specifically, individuals with greater addiction tendencies may exhibit reduced cognitive resources for managing conflict during incongruent trials, thereby manifesting a diminished theta power difference in EEG measurements. Should future endeavors explore neuroregulatory interventions for mobile phone short video addiction, theta difference power may hold promise as a crucial neural reference index.

Notably, the most important result was found between MPSVATQ and neural oscillation index calculated by subtracting the theta power of neutral condition from that of incongruent target, which was used to characterize individuals’ ability of executive control. However, in previous studies, executive control in ANT was usually defined by the difference between the incongruent and congruent conditions, even in the calculation of EEG indicators (Neuhaus et al., 2010; Racer et al., 2011; Chang et al., 2015). In the current study, the reaction time of the congruent condition was slightly longer than that of the neutral condition, but did not show statistically significant difference. It is plausible to postulate that the neutral condition functions as a baseline, involving solely the processing of the central arrow’s direction, without the need to suppress any conflicting stimuli. In the congruent condition, even though the direction of the central arrow aligns with that of the surrounding arrows, there remains a requirement to divert attention to minimize the impact of the adjacent arrows. This indicates that, similar to what has been observed in the incongruent condition in prior research, attentional processing demands are still present in the congruent condition (Appelbaum et al., 2011). The significant correlation between MPSVATQ and neural oscillation index calculated by subtracting the theta power of neutral condition from that of incongruent target, but not with that calculated by subtracting the theta power of congruent condition from that of incongruent target further confirmed this argument. However, the specific neurophysiological implication warrants further study.

Finally, this study found a significant negative correlation between short video addiction tendency and self-control questionnaire scores, aligning with prior research emphasizing the relationship between addictive behavior and self-control difficulties (Racer et al., 2011; Blachnio and Przepiorka, 2016). This underscores the importance of addressing self-control enhancement in interventions targeting short video addiction. Numerous studies have shown that mindfulness meditation is associated with reduced addictive behaviors (Bowen and Vieten, 2012; Davis et al., 2014a,b; Sriburapar, 2021; Parisi et al., 2023). It effectively reduces the risk of Internet addiction by enhancing an individual’s level of self-control. This increase in self-control further leads to a decrease in the individual’s stress level, which reduces the likelihood of developing Internet addiction (Song and Park, 2019). It can be inferred that mindfulness training may also have a positive effect on reducing addiction to mobile phone short-form videos. No significant result was observed when assessing the relationship between self-control and theta activity in the frontal area reflecting three sub-networks assessed during ANT paradigm. Since self-report measures of trait self-control capture central tendencies of aggregates of many different instances of behavior, whereas behavioral inhibition tasks are momentary one-time state measures (Wennerhold and Friese, 2020). These results indicate that the theta power difference between incongruent and neural target conditions primarily captures a momentary state rather than a comprehensive measure of self-control.

## 5 Conclusion

The present study was undertaken to delve into the association between mobile phone short video addiction tendency and attentional functions, as well as potential changes in brain activity. We found a significant negative correlation between short video addiction tendency and neural oscillation index reflecting the executive control network in the frontal area. This finding suggests that a higher propensity for short video addiction may impair executive control. Furthermore, we identified a strong negative correlation between short video addiction and self-control abilities, indicating that higher addiction levels are associated with diminished self-control. Short-form videos, being self-stimulating and content-rich, capture attention with minimal psychological effort. Prolonged consumption of such content may primarily engage the lower-order cortical brain regions, such as those associated with emotional processing, and suppress activity in higher-order areas responsible for self-control and attention. This pattern may enhance the susceptibility to short video addiction, with a simultaneous decline in self-control. Given the current findings, we can develop mental training interventions to enhance self-control, for example, incorporating mindfulness meditation practices to address self-control deficits associated with short video addiction.

## 6 Limitations and recommendations for future research

Although this study has yielded findings, it is not without its limitations. To further delve into the cognitive mechanisms of smartphone short video addiction, several suggestions for future research are proposed. Firstly, longitudinal research designs can be employed to track the dynamics of individual addiction tendencies and cognitive functions, unraveling causal relationships. Secondly, future studies could benefit from a larger and more diverse sample size, including a wider range of participants such as children and the elderly. Additionally, employing a more gender-balanced sample and extending the research to encompass a greater variety of short video categories, like those found on YouTube, would enhance the generalizability of the current findings. Thirdly, the incorporation of neuroimaging techniques, such as functional magnetic resonance imaging (fMRI), could enable more precise delineation of brain activity alterations. Fourthly, exploring additional factors influencing smartphone short video addiction, such as daily mobile short video usage time, social and psychological variables, would contribute to a more comprehensive understanding of psychological mechanisms of short video addiction. Lastly, future research could also look into developing and testing interventions based on the findings of this and similar studies, aiming to mitigate the effects of short video addiction on attentional functions.

## Data availability statement

The original contributions presented in this study are included in this article/Supplementary material, further inquiries can be directed to the corresponding authors.

## Ethics statement

The studies involving humans were approved by the Ethics Committee, Department of Psychology and Behavioral Sciences, Zhejiang University. The studies were conducted in accordance with the local legislation and institutional requirements. The participants provided their written informed consent to participate in this study.

## Author contributions

TY: Conceptualization, Data curation, Investigation, Methodology, Writing – original draft, Writing – review & editing. CS: Conceptualization, Formal analysis, Writing – review & editing. WX: Conceptualization, Formal analysis, Software, Writing – review & editing. YH: Funding acquisition, Methodology, Supervision, Writing – original draft, Writing – review & editing. HZ: Funding acquisition, Supervision, Writing – review & editing.

## Footnotes

1
http://psychtoolbox.org/

2
http://sccn.ucsd.edu/eeglab
