# Supplementary material for: Mobile phone short video use negatively impacts attention functions: an EEG study
Source: Front Hum Neurosci. 2024 Jun 27;18:1383913. doi: 10.3389/fnhum.2024.1383913 (PMC11236742; doi:10.3389/fnhum.2024.1383913)
Supplement: Supplementary file 1 [file Data_Sheet_1.PDF]

Supplementary Materials

Experimental Protocol

This research is part of a broader project investigating the effects of short-form video consumption on brain function and structure. sFigure 1 schematically illustrates the complete experimental protocol. Participants first completed an online questionnaire survey. Next, they started with a 3-minute resting state with eyes open, followed by the Attention Network Test (ANT), and concluded with another 3-minute resting state with eyes open. Participants then completed a fixed-duration video-watching task, during which they watched 5-second clips of various short videos and rated their preference and desire for each clip on a 1-5 point scale. After this task, they transitioned to another 3-minute resting state with eyes open. During the subsequent free viewing of a series of short videos, participants could switch to the next video if they found the current one unappealing. Upon completing the free viewing phase, participants answered five questions about their emotional state, responding on a 1-5 point scale. Finally, they entered one last 3-minute resting state with eyes open.

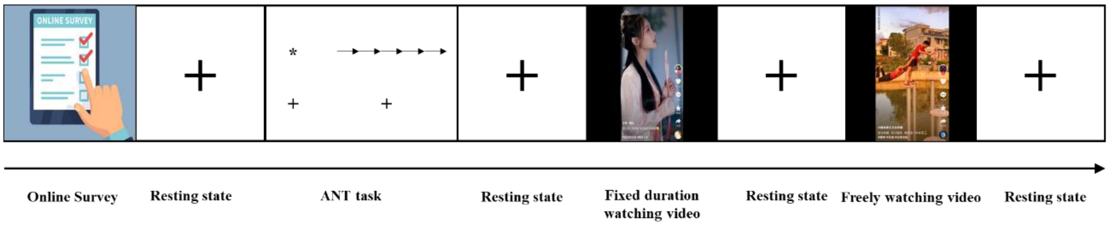

sFigure 1. Schematic representation of the experimental protocol

Behavior results

sTable1. Results of correlation analysis among the 10 questionnaires (*N* = 48)

	MPSVTQ	Anxiety	Depression	IAT	BIS-II	CPPS	SCS	MWQ	ACS	FFMQ
MPSVTQ	-	-	-	-	-	-	-	-	-	-
Anxiety	.151	-	-	-	-	-	-	-	-	-
Depression	.103	.518**	-	-	-	-	-	-	-	-
IAT	.390**	.377*	.313	-	-	-	-	-	-	-
BIS-II	-.078	.165	.211	-.324*	-	-	-	-	-	-
CPPS	-.149	.312*	.542**	.466**	.492**	-	-	-	-	-
SCS	-.320*	-.274	-.254	-.726**	-.422**	-.423**	-	-	-	-
MWQ	.231	.314*	-.306*	.619**	.303*	.340*	-.586**	-	-	-

**ACS**	-.310*	-.292*	-.152	-.433**	-.156	-.431**	.268	-.353*	-	-
**FFMQ**	-.103	-.203	-.413**	-.300	-.428**	-.562**	.115	-.233	.252	-

（$*p < 0.05$, $**\ p < 0.01$, $***\ p < 0.001$)

sTable 2. Correlation between MPSVTQ and RT of ANT's Three Sub-networks (*N* = 48)

ANT function	Cue/Target	*R*	*P*
**Alerting**	No cue-Double cue	-0.035	0.812
**Orienting**	Center cue-Spatial cue	0.198	0.177
**Inhibition**	Incongruent target-Congruent target	-0.153	0.299
	Incongruent target-Neutral target	0.037	0.805

（$*\ p < 0.05$, $**\ p < 0.01$, $***\ p < 0.001$)

In the sTable 2, no significant correlation was found between the MPSVTQ and the RT performance of the three subnetworks of the ANT. And there is no significant difference in RT between congruent target and neutral target ($t\ (43) = -1.894$, $p = 0.064 > 0.05$).

EEG results

sTable 3. Correlation coefficients between MPSVATQ and theta power indexes of ANT's three subnetworks (*N* = 45)

ANT Function	Cue/Target	Brain Regions	*R*	*P*
**Alerting** (650-800ms)	No cue-Double cue	Parietal region	-0.134	0.379
		Parietal Occipital region	-0.061	0.692
		Occipital region	0.001	0.995
**Orienting** (600-800ms)	Center cue-Spatial cue	Parietal region	-0.072	0.649
		Parietal Occipital region	-0.027	0.863
		Occipital region	-0.078	0.623
**Executive control** (600-900ms)	Incongruent target-Congruent target	Frontal region	-0.151	0.323
		Middle Frontal region	-0.133	0.383
		Central region	-0.143	0.347

	Parietal region	0.147	0.335
	Parietal Occipital region	0.230	0.128

（* $p < 0.05$, ** $p < 0.01$, *** $p < 0.001$)

No notable correlation was observed between MPSVATQ scores and theta power across the three ANT subnetworks in distinct brain regions.

sTable 4. Correlation coefficients between MPSVATQ and theta power difference between incongruent target and neutral target (after adding covariates) ($N = 45$)

Control variable	Brain Region	*R*	*p*	*df*
**Gender & Age & Anxiety & Depression**	**Frontal** region	**-0.429****	**0.005**	39
	Middle Frontal region	-0.324*	0.039	39
	Central region	-0.273	0.084	39
	Parietal region	-0.012	0.941	39
	Parietal Occipital region	-0.127	0.430	39

（* $p < 0.05$, ** $p < 0.01$, *** $p < 0.001$)

After incorporating control variables such as gender, age, anxiety, and depression, a noteworthy correlation persisted between the MPSVATQ and theta power in the frontal area under incongruent target minus neutral target.

sTable 5. Correlation coefficients between SCS and theta power difference between incongruent target and neutral target ($N = 45$)

Brain Regions	*R*	*p*
**Frontal** region	0.253	0.093
Central-Frontal region	0.195	0.199
Central region	-0.037	0.808
Parietal region	-0.113	0.462
Parietal Occipital region	-0.037	0.809

（* $p < 0.05$, ** $p < 0.01$, *** $p < 0.001$)

No notable correlation was observed between the SCS and theta power in distinct brain regions under incongruent target minus neutral target.

sTable 6. Correlation: MPSVTQ and theta power in the resting state before and after ANT (*N* = 45)

**Brain regions**	**Rest**	***R***	***P***
Frontal region	Before ANT	-0.033	0.825
	After ANT	-0.170	0.254

No significant correlation was observed between the MPSVTQ and theta power in the resting state, both before and after the ANT.
